# Resolving protein condensates and aggregates in vivo by boxcar-enhanced Fluorescence-detected mid-Infrared photothermaL Microscopy (FILM)

**DOI:** 10.1038/s42004-026-01970-3

**Published:** 2026-05-11

**Authors:** Bethany Weinberg, Zhongyue Guo, Rong Tang, Jianpeng Ao, Jiaze Yin, Guangrui Ding, Todd A. Blute, Marzia Savini, Giulio Chiesa, Wentao Wang, Matt Good, Meng C. Wang, Ji-Xin Cheng

**Affiliations:** 1 Department of Molecular Biology, Cell Biology, & Biochemistry, Boston University, Boston, MA USA; 2 Department of Biomedical Engineering, Boston University, Boston, MA USA; 3 Department of Electrical and Computer Engineering, Boston University, Boston, MA USA; 4 Photonics Center, Boston University, Boston, MA USA; 5 HHMI Janelia Research Campus, Ashburn, VA USA; 6 Department of Biomedical Engineering, University of Toronto, Toronto, ON Canada; 7 Cell and Developmental Biology, School of Medicine, University of Pennsylvania, Philadelphia, PA USA

**Keywords:** Chemical tools, Protein aggregation

## Abstract

Protein assemblies, including aggregates and condensates, are closely linked to health and diseases. We demonstrate boxcar-enhanced Fluorescence-detected mid-Infrared photothermaL Microscopy (FILM), using two model species, *Caenorhabditis elegans* and *Saccharomyces cerevisiae*, to quantitatively resolve these protein states in vivo by imaging β-sheet and α-helix secondary structures and analyzing their ratios. This method directly distinguishes polyglutamine (PolyQ) protein aggregates, α-synuclein protein condensates, and P-granule condensates implicated in neurodegenerative diseases and embryonic development in live organisms. It further enables the unraveling of protein assembly dynamics and their physio-pathological roles, such as age-related progression of PolyQ from condensates to aggregates.

**Figure:**
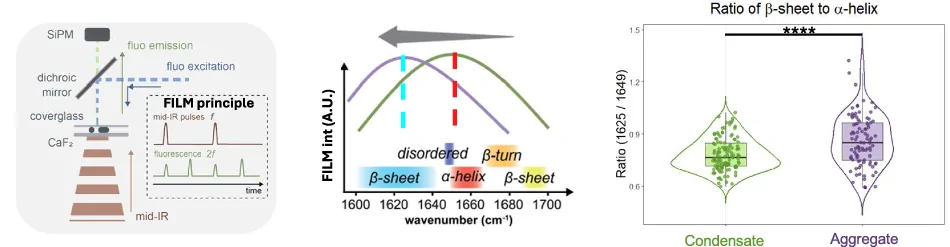

## Abstract (web summary)

Protein assemblies — either aggregates or condensates — exhibit distinct β-sheet, α-helix, or disordered structures; however, current fluorescence-based approaches do not provide information on such secondary structures. Here, the authors show that boxcar-enhanced Fluorescence-detected mid-Infrared photothermaL Microscopy (FILM) can be used to quantitatively resolve these protein states, providing a non-invasive approach to directly distinguish between protein condensates and aggregates in vivo.

## Introduction

Proteins are one of the most structurally diverse biomolecules within the cell. Proteins can form larger assemblies, serving distinctive physiological and pathological roles. Rigid, insoluble protein aggregates are widely observed in neurodegenerative diseases, where they contribute to disease progression^1^. On the other hand, liquid-like protein condensates, which form membrane-less organelles through phase separation, play critical roles during specific developmental stages and dynamically adapt to cellular responses^2^. Pre-existing methods for studying aggregates and condensates rely on conventional fluorescence microscopy to assess protein fluidity through fluorescence recovery after photobleaching (FRAP)^3^. For instance, FRAP analysis showed that poly-glutamine (PolyQ) aggregates do not recover from bleaching even after 5 min^4^, whereas proteins within condensates in *Caenorhabditis elegans* recover within just one second^5^. However, bleaching is low-throughput and may disturb or destabilize protein assemblies, especially those with sensitive structures. Moreover, this approach does not provide any information about the secondary structures of fluorescently-labeled protein assemblies.

**Fig. 1:**
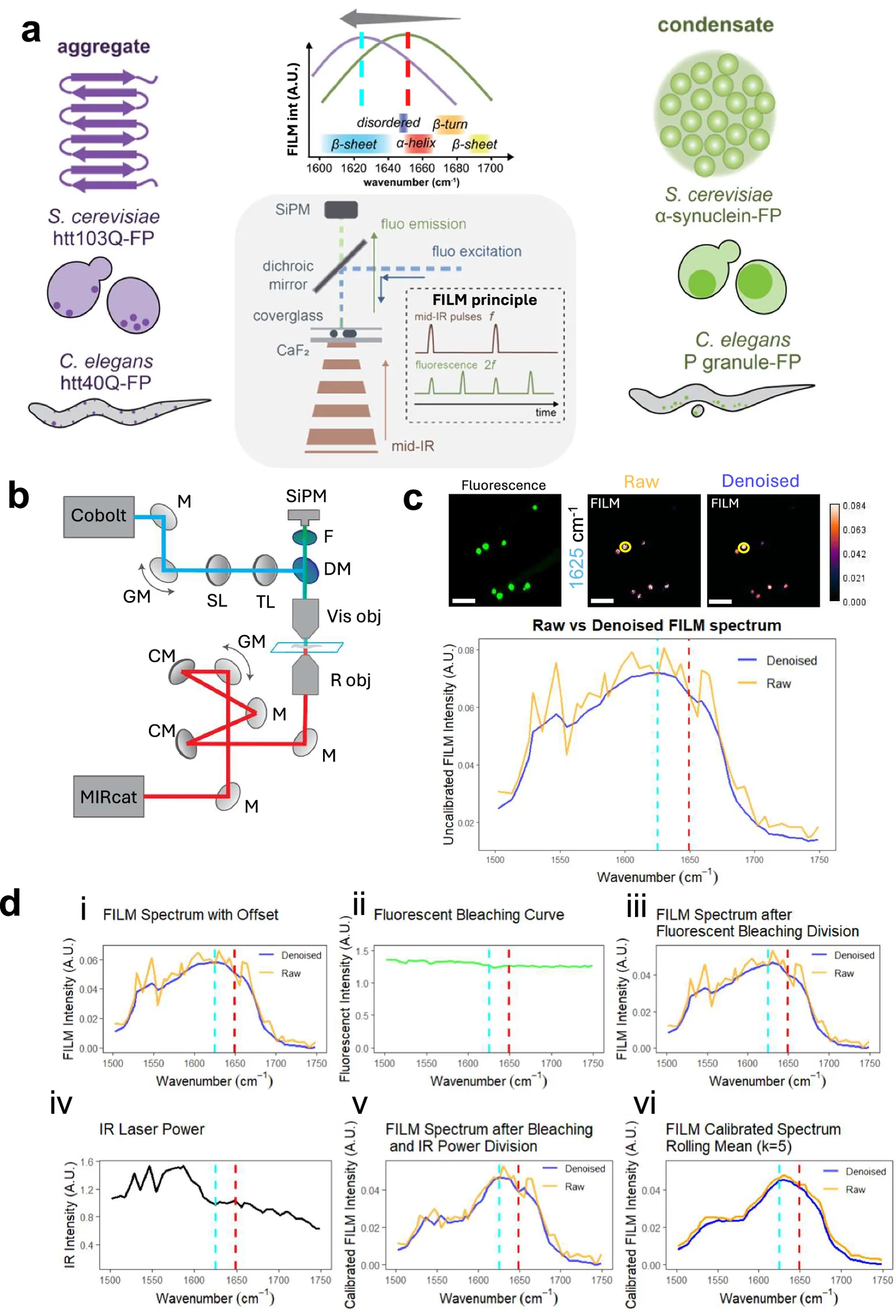
Boxcar-enhanced FILM concept, microscope, denoising, and calibration workflow. **a** Concept of boxcar-enhanced FILM for differentiating aggregates (purple, left) and condensates (green, right) in yeast and worm models. β-sheets have a peak at 1625 cm^−1^, while α-helices have a peak at 1649 cm^−1^, which can be detected using boxcar-enhanced FILM (gray panel). **b** Schematic of the experimental setup for the boxcar-enhanced FILM microscope. Red: IR laser. Blue: visible excitation. M reflection mirrors, GM galvo mirrors, CM concave mirrors, SL scan lens, TL tube lens, DM dichroic mirror, Vis obj visible objective, R obj reflective objective, F filter, SiPM silicon photomultiplier. **c** Representative images of fluorescence, raw boxcar-enhanced FILM, and SPEND-denoised boxcar-enhanced FILM signal (top panels). The spectrum for the region highlighted in yellow in both the raw boxcar-enhanced FILM (orange) and denoised (blue) signal are shown for comparison (bottom panel). Scale bar 20 µm. **d** Representative spectra for calibration protocol, compared between the raw and denoised spectrum for the region highlighted in yellow in (**c**). i Boxcar-enhanced FILM spectra with an offset applied to bring the lowest value to 0. ii The fluorescent bleaching curve obtained from the fluorescent image in (**c**). iii Boxcar-enhanced FILM spectra after division by bleaching curve (i/ii). iv The IR laser power obtained at the sample plane for the Wavenumbers of interest. v Boxcar-enhanced FILM spectra after division by IR laser power (iii/iv). vi Calibrated spectra after 5 points of smoothing. Note that denoising does not impact spectrum shape or peaks of interest.

A key component associated with protein assemblies is their secondary structure. Protein aggregates are typically rich in β-sheet secondary structures due to a network of stable intermolecular hydrogen bonds, while condensates are associated with α-helices or disordered secondary structures that enable dynamic interactions^6,7^. Notably, IR absorption at the amide I region (~1600–1700 cm^−1^) is sensitive to protein secondary structures. For example, Aβ fibrils (a type of protein aggregates found in Alzheimer’s Disease) formed in primary neurons exhibit a shift in their IR spectrum towards 1630 cm^−1^, which is associated with β-sheet structures^8^. PolyQ aggregates in *Saccharomyces cerevisiae* are composed of a β-sheet core surrounded by an α-helix shell. The β-sheet core exhibits a peak shift toward 1625 cm^−1^, whereas the α-helix shell retains its peak at 1650 cm^−1^ ^9^. We thus hypothesized that probing the secondary structures of protein assemblies would provide a non-invasive approach to directly distinguish between protein condensates and aggregates in vivo. In addition, we hypothesized that aggregation of PolyQ may exhibit age-dependent secondary structure changes undetected by traditional fluorescent visualization. We employed boxcar-enhanced FILM^10^ (Fig. 1a) to determine the secondary structure of fluorescently-labeled protein assemblies.

Various vibrational spectroscopic imaging methods, both Raman and IR-based, have been developed to study protein secondary structure^11^. Stimulated Raman scattering (SRS) microscopy was recently used to track phase separation of condensates and Htt aggregation in situ^12^. BonFIRE enabled single-bond vibrational spectroscopy of fluorescent molecules based on up-conversion^13^. Mid-infrared photothermal microscopy could distinguish shell vs core aggregate secondary structure in *S. cerevisiae* Huntington models, where spectral deconvolution was used to obtain component secondary structure peaks^9^. The current work, utilizing boxcar-enhanced FILM, enables spectroscopic peak assignments for α-helices and β-sheets obtained directly from hyperspectral stacks of images. We demonstrate the use of boxcar-enhanced FILM to quantitatively distinguish between condensates and aggregates using two peaks associated with secondary structure, while also demonstrating our method’s ability to distinguish between the secondary structure heterogeneity found between condensates and across aggregate ages in vivo.

Boxcar-enhanced FILM combines mid-IR to probe vibrational modes of biomolecules with the use of a fluorescent reporter, as seen with recently developed FILM (also called F-MIP)^14,15^. When the mid-IR wavenumber matches that of the vibrational mode of a biomolecule, such as C=O at 1650 cm^−1^ for proteins, the molecule absorbs energy and then releases it as heat into the surrounding environment. As fluorescence intensity decreases upon exposure to heat, we can use a modulated mid-IR pulse and track changes in fluorescence intensity as readout for biomolecules. This enables a localized analysis of fluorescently-tagged condensates and aggregates. However, photobleaching can negatively affect results in FILM. To override this limitation, an optical-boxcar scheme is applied to effectively reduce bleaching with a pulsed fluorescent probe to limit excitation of the fluorophore. If the mid-IR pulse is at frequency *f*, the visible pulse is at frequency 2 *f*; in the presence of a biomolecule, fluorescence intensity is decreased when the mid-IR pulse is on (*f*) and brighter when mid-IR is off (2 *f*), the modulation of which generates the FILM signal. Rather than replacing the lock-in amplifier with a boxcar averager, as reported by the Sander group^16^, we instead engineered the optical excitation by converting continuous-wave (cw) light into pulses. This approach inherently shifts higher-order frequency components into the detection band while simultaneously reducing the photodose delivered to the sample, thereby mitigating fluorescence photobleaching^10^. Finally, boxcar-enhanced FILM is combined with a laser-scan scheme that enables hyperspectral imaging in the fingerprint window (1000–1800 cm^−1^)^10,17^ (Fig. 1b).

In addition to the instrumental advance, we also applied a self-supervised denoising AI framework, Self-supervised PErmutation Noise2noise Denoising (SPEND)^18^. Traditional denoising methods, such as BM4D, assume independent and identically distributed (i.i.d.) noise throughout the image, but hyperspectral data contains noise that is spatially-correlated, complicating the selection of denoising algorithms. SPEND overcomes these challenges by using even and odd spectral frames to generate two stacks with the same levels of noise. These two stacks can then be used for self-supervised noise-to-noise training, which can generate an 8-fold signal-to-noise ratio (SNR) improvement when the model is then applied to the original hyperspectral stack (Fig. 1c). In addition, we present a calibration protocol to apply to denoised spectra to account for fluorescent bleaching and IR laser power distortions (Fig. 1d).

We have validated our method in two testbeds, *S. cerevisiae* and *C. elegans*, as shown below. Overall, our work reveals the dominant secondary structure and heterogeneity of condensates. We also show age-related progression of PolyQ Huntington protein assemblies into β-sheet dominant aggregates over time.

## Results and discussion

### Quantification of *S. cerevisiae* condensate and aggregate secondary structure

**Fig. 2:**
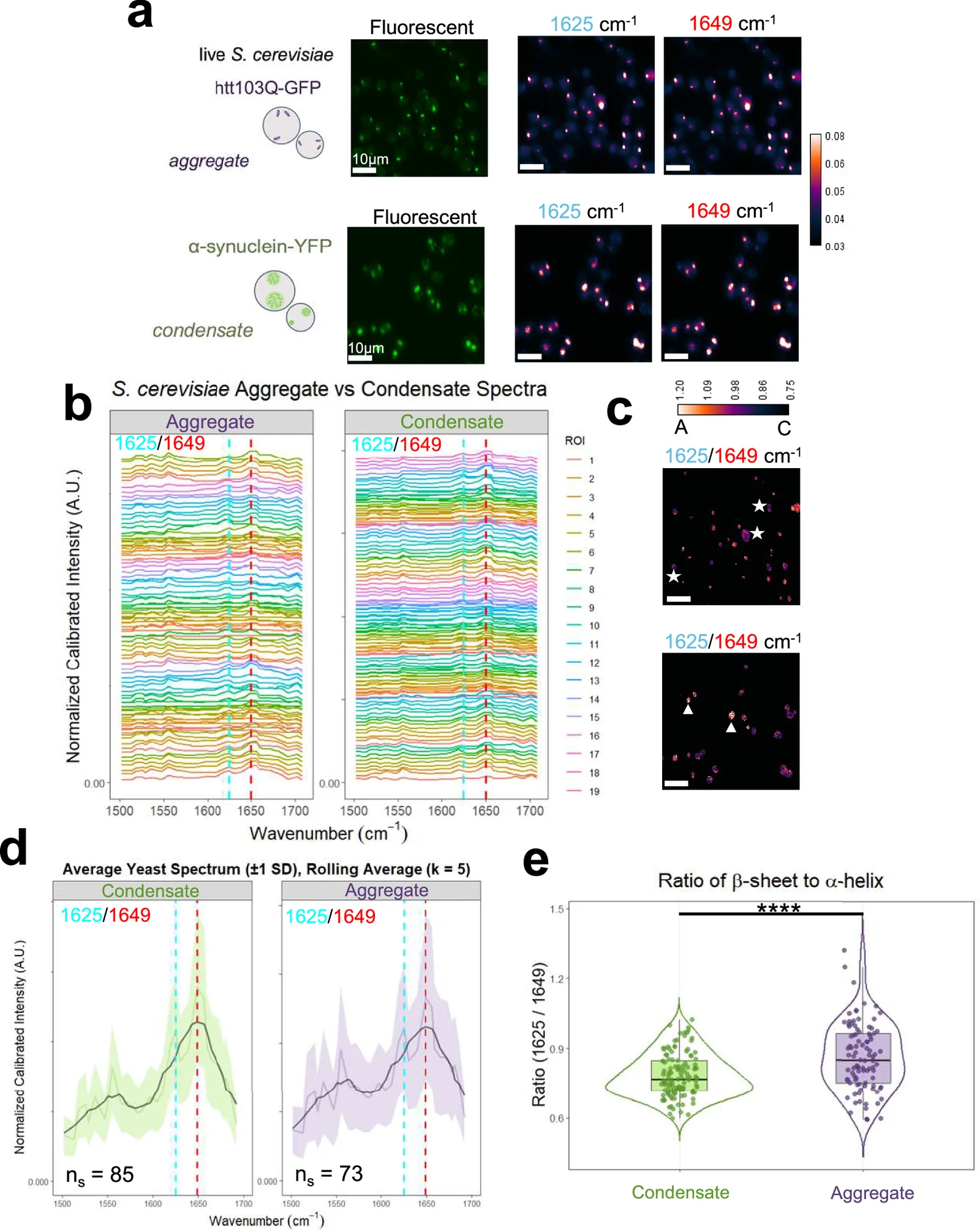
Boxcar-enhanced FILM differentiates condensates and aggregates in-vivo in yeast. **a** Fluorescent and boxcar-enhanced FILM-acquired images of *S. cerevisiae* expressing aggregate (htt103Q) and condensate (α-synuclein). The fluorescent images (left panels) and boxcar-enhanced FILM acquired images at 1625 and 1649 cm^−1^ are shown. Scale bar 10 µm. **b** Calibrated *S. cerevisiae* aggregate and condensate spectra included in analysis. **c** Ratiometric image generated using a masked division of images for regions with signal shown in (**a**). Scale bar 10 µm. **d** Average and smoothed spectrum obtained from all spectra in (**c**). n_s_ (number of spectra) = 85 for condensates, 73 for aggregates. **e** 1625/1650 cm^−1^ ratio analysis comparing condensates (α-synuclein-FP) and aggregates (Htt103Q-FP) in yeast cells. ****p value < 0.0001 by pairwise Student’s *t-*test.

We first examined *S. cerevisiae* strains carrying well-defined protein aggregates and condensates (Fig. 2a). *S. cerevisiae* htt103Q-FP strain contains GFP tagged with 103 glutamine repeats (PolyQ), which form small, rigid aggregates like those seen in Huntington’s Disease. On the other hand, *S. cerevisiae* α-syn-FP strain contains YFP-tagged α-synuclein, which exhibits larger, dispersed structures commonly used for studying condensates (Fig. 2a, fluorescent). We used boxcar-enhanced FILM to image individual htt103Q-FP and α-syn-FP protein assemblies in yeast cells at the peaks associated with β-sheets (1625 cm^−1^) and α-helices (1649 cm^−1^) (Fig. 2a, right panels), as well as capture their spectra that cover the entire amide I and amide II range (1450–1750 cm^−1^) (Fig. 2b and Supplementary Fig. 1a). After SPEND-mediated denoising, we calculated the ratio between the signal intensity at 1625 and 1649 cm^–1^. We found that htt103Q-FP aggregates exhibit a higher ratio than α-syn-FP condensates (Fig. 2d, e), supporting a higher proportion of β-sheet secondary structure in aggregates compared to condensates. At the same time, we observed htt103Q-FP aggregates with a lower proportion of β-sheets (stars) and α-syn-FP condensates with a higher proportion of β-sheets (arrowheads), suggesting a continuous progression from condensates to aggregates in vivo (Fig. 2c). The high-throughput, non-invasive analysis enabled by boxcar-enhanced FILM allows for the examination of a large number of protein assemblies and quantitative characterization of their structural states in vivo.

### Resolution of age-related shifts toward PolyQ aggregates in *C. elegans*

**Fig. 3:**
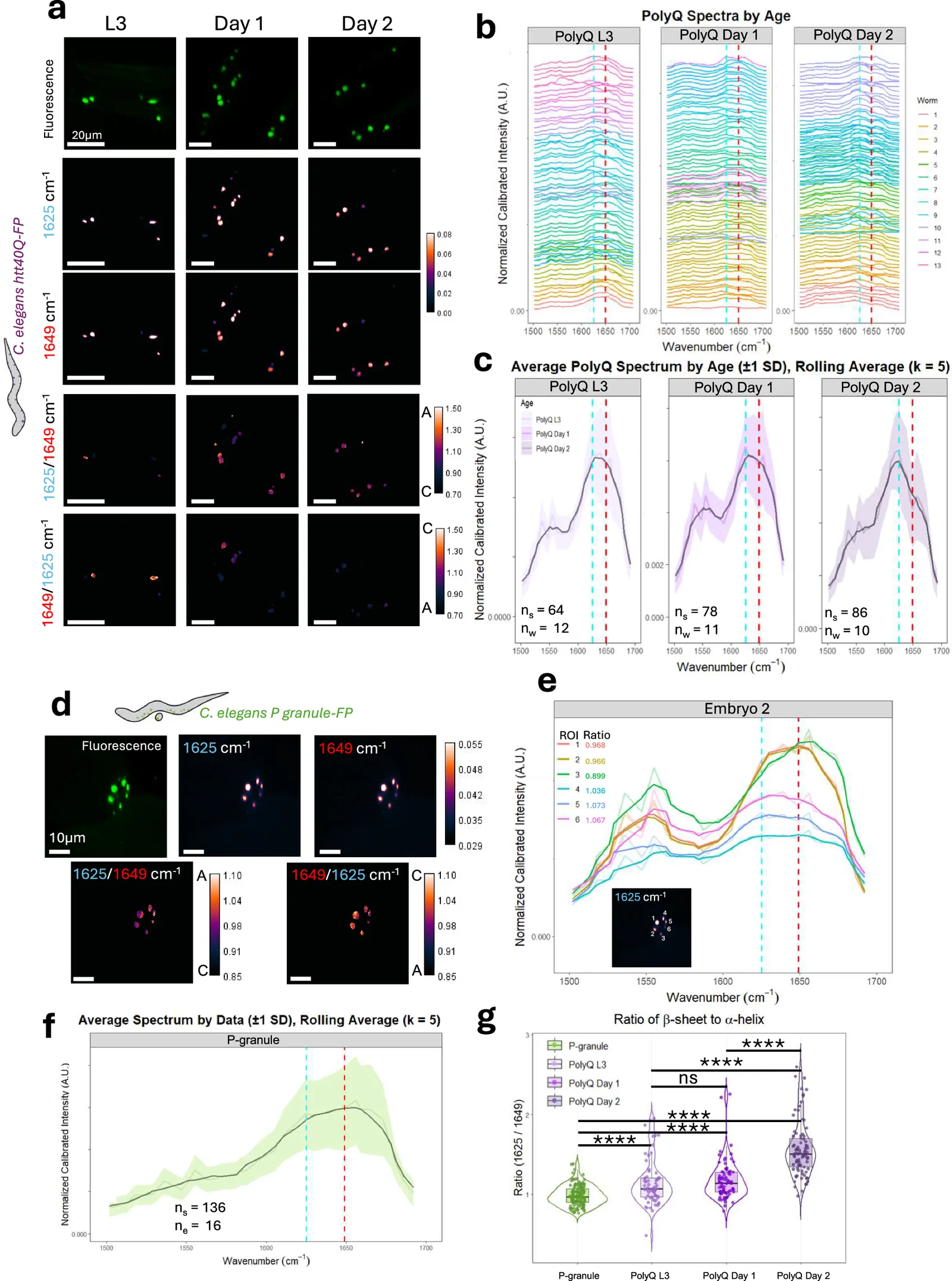
Age-related secondary structure changes revealed by boxcar-enhanced FILM in live worms. **a** Representative images of fluorescent and boxcar-enhanced FILM images of PolyQ (htt40Q) *C. elegans* at L3 (larval stage 3), day 1, and day 2 adults. Ratiometric images were generated using a masked division of images for regions with signal. Scale bar 20 µm. **b** Calibrated *C. elegans* aggregate (PolyQ) spectra included in analysis. **c** Average and smoothed spectrum obtained from all spectra in (**b**). *n*_s_ (number of spectra) = 64 for L3, 78 for PolyQ day 1, and 86 for PolyQ day 2. *n*_w_ (number of worms) = 12 for L3, 11 for day 1, and 10 for day 2. **d** Representative images of fluorescent and boxcar-enhanced FILM condensates (P-granules). Ratiometric images were generated using a masked division of images for regions with signal. Scale bar 10 µm. **e** Representative amide I and II calibrated spectra of selected regions found in embryo 2. **f** Average P-granule spectrum obtained from all spectra in Supplementary Fig. 2a. *n*_s_ (number of spectra) = 136. *n*_e_ (number of embryos) = 16. **g** 1625/1650 cm^−1^ ratio analysis comparing all ages of PolyQ (aggregate) worms to embryo P-granule condensates. ****p value < 0.0001 by pairwise Student’s *t*-test.

Next, we applied boxcar-enhanced FILM to examine two *C. elegans* strains that carry either fluorescent protein aggregates (httQ40-FP) or condensates (P-granule-FP) (Fig. 1a). As with yeast htt103Q, YFP-tagged 40 PolyQ forms aggregates in the muscle of the worm^19^. We imaged the strain at L3 (larval stage 3), day 1, and day 2 adults, and examined whether the secondary structure of httQ40::YFP aggregates varies with increasing age (Fig. 3a). We observed that the signal of httQ40::YFP puncta at the 1649 cm^−1^ peak, corresponding to α-helices, is higher in day 1 adults than in day 2 adults, whereas the signal at the 1625 cm^−1^ peak, corresponding to β-sheet, is lower (Fig. 3a–c). Interestingly, the signal at the 1625 cm^−1^ peak in L3 larvae is lower than that observed in day 1 and day 2 adults (Fig. 3c), while the signal at the 1649 cm^−1^ peak is comparable between L3 and day 1 but lower in day 2 adults. These results suggest that the proportion of α-helices in httQ40 protein assemblies decreases with age, while the assemblies adopt more β-sheet-enriched aggregate structures. Boxcar-enhanced FILM can quantitatively track this age-related change in a high-throughput and high-resolution matter in live organisms (Fig. 3g).

### Visualization of secondary-structural heterogeneity within *C. elegans* P-granule condensates

GLH-1 is a *C. elegans* helicase that facilitates the formation of P-granules, which are phase-separated condensates in germ cells^20^. Using the GLH-1::GFP strain (P-granule-FP), we imaged P-granules in multiple embryos at various developmental stages using boxcar-enhanced FILM (Fig. 3d and Supplementary Fig. 2a). We observed that those protein condensates, even within the same egg, display heterogeneity in their spectra (Fig. 3e). We further calculated the ratio of 1625–1649 cm^−1^ for each GLH-1::GFP condensate. We found that the average ratio is lower than that of httQ40::YFP puncta in day 1 and day-2-old adults, but comparable to that of httQ40::YFP puncta in L3 larvae (Fig. 3f, g and Supplementary Fig. 2b, c). These results suggest that httQ40::YFP assemblies may initially form as liquid-like condensates and progressively transition into aggregate structures during aging.

### Validation of age-related PolyQ aggregation using FRAP

**Fig. 4:**
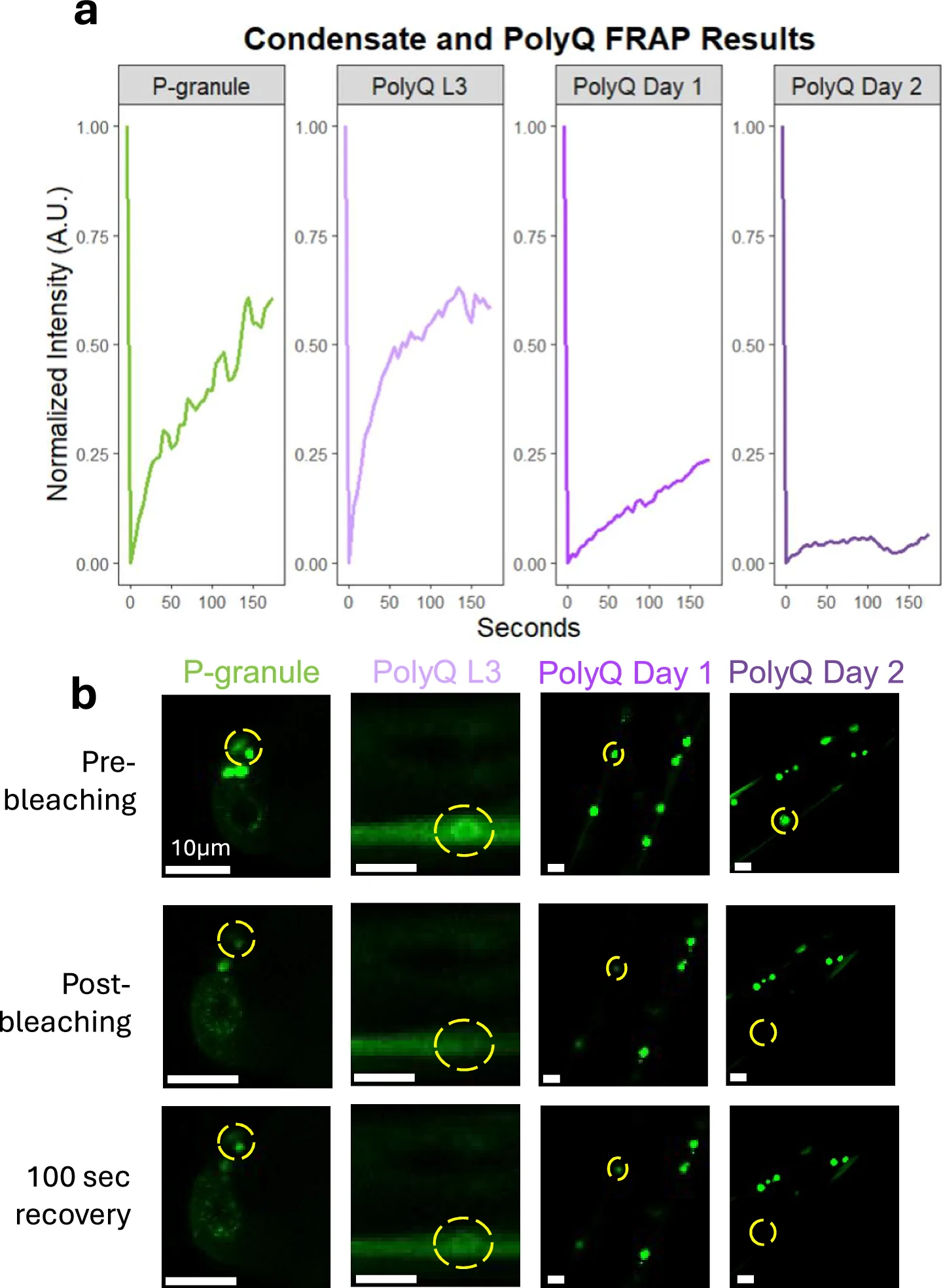
Validation of boxcar-enhanced FILM results by FRAP measurements. **a** Fluorescence-recovery after photobleaching recovery curves for *C. elegans* embryonic P-granules, and L3, day 1, and day 2 adult PolyQ aggregates. **b** Images showing the region bleached and used for the graphs in (**a**) highlighted in yellow. The PolyQ assemblies shown to contain high β-sheet with boxcar-enhanced FILM (Fig. 3g) display the slowest FRAP recovery. Scale bar 10 µm.

Finally, we compared our aggregate and condensate boxcar-enhanced FILM results obtained from *C. elegans* to that of FRAP (Fig. 4a, b). Our results indicate that P-granule condensates and L3 PolyQ puncta have comparable recovery curves, while day 2 PolyQ aggregates had little to no recovery. Day 1 PolyQ puncta showed an intermediate recovery speed. This FRAP data matches well with our boxcar-enhanced FILM data (Fig. 3g); P-granules and L3 PolyQ puncta contained the lowest proportion of β-sheets and showed the fastest FRAP recovery time, while day 2 PolyQ aggregates contained the highest ratio of β-sheets and showed the slowest FRAP recovery time. FRAP requires bleaching aggregates and condensates and waiting for their recovery one by one, which limits its throughput and its quantitative capability. In contrast, boxcar-enhanced FILM can analyze multiple protein assemblies simultaneously within a short time window, enabling robust quantitative comparisons across conditions.

## Conclusions

Together, our findings revealed that protein aggregates and condensates exhibit different secondary structures, and we demonstrated a non-invasive method to quantitatively distinguish them in live cells and organisms using boxcar-enhanced FILM. This method enables rapid, high-throughput survey of large numbers of protein assemblies to determine their in vivo states. In a proof-of-concept application to Huntington-disease models, we discovered that PolyQ proteins exhibit heterogenous structural states despite their similar fluorescence morphology, and progress into β-sheet dominant protein aggregates over time. Future integration of this method with correlated cryo-electron tomographic microscopy will help validate the secondary-structure features of these protein assemblies detected by boxcar-enhanced FILM, and fully solidify its utility in structure biology. This method can be readily applied to other aggregate and condensate models that contain fluorescent proteins, paving the way for tracking their temporal and spatial dynamics under various physiological and pathological conditions.

## Methods

### Boxcar-enhanced FILM

The microscope set-up described has been published in 10; please see that paper for a detailed overview of boxcar-enhanced FILM. Briefly, the pulsed mid-infrared (IR) pump beam is generated by a wavelength-tunable quantum cascade laser (QCL, Daylight Solutions, MIRcat-QT-Z-2400). Fluorescence excitation light is provided by a 488 nm fixed-wavelength diode laser module (Cobolt, 06-MLD 488 nm), which can be digitally modulated into pulsed light via an external trigger. A function generator synchronizes the visible excitation light and the mid-IR pump beam, with their modulation frequencies set to 2 *f* (400 kHz) and *f* (200 kHz), respectively. The IR pulse width is set from anywhere between 200 ns and 500 ns, depending on the sample, while the visible light operates with a 30% duty cycle. The fluorescence excitation light is rapidly scanned using a pair of dual-axis galvo mirrors (GVS002, Thorlabs). After passing through a scan lens (*f* = 100 mm; a pair of AC508-100-A, Thorlabs) and a tube lens (*f* = 200 mm; TTL200-A, Thorlabs), the beam is reflected by a dichroic mirror (DM) into a water-immersion objective (UPlanSApo, Olympus, 60×, NA = 1.2) and focused onto the sample. The IR beam is scanned independently with another pair of X-Y galvanometer mirrors (GVS002, Thorlabs). The IR beam path employs a concave mirror as the scan lens (*f* = 200 mm; CM508-200-P01, Thorlabs) and a tube lens (*f* = 500 mm; CM508-500-P01, Thorlabs) to relay the scan to the back pupil of a reflective objective (PIKE, 40×, NA = 0.78), achieving counter-propagation alignment with the visible excitation light. Before imaging, the IR beam is carefully aligned to overlap with the visible focus.

During imaging, the IR and visible foci are synchronously scanned, ensuring uniform excitation and detection over the FOV. The two galvanometer pairs are synchronized with the focal lengths of the visible and IR objectives and scaled based on the beam expansion ratio of the relay system. This scaling factor is calibrated at the start of the experiment. The backward fluorescence emitted from the sample is collected by the water-immersion objective and directed through the DM. After further filtering with a bandpass filter, the fluorescence signal is detected by a silicon photomultiplier (SiPM, Hamamatsu, C13366-3050GA). The resulting electrical signal is fed into Moku:Pro (Liquid Instrument, Multi-instrument Mode), filtered, and input into the slots of two lock-in amplifiers for demodulation at 2*f* and *f* frequencies, corresponding to the FILM and fluorescence DC signals, respectively. These demodulated signals are simultaneously acquired through two input ports of an acquisition card, enabling real-time dual-channel imaging.

To perform hyperspectral imaging, the QCL operates in multi-spectral mode using a preset scanning list that covers the range from 1000 to 1800 cm^−^¹. A total of 126 Wavenumbers were collected in the fingerprint region (1000–1800 cm^−1^).

### Denoising with self-supervised permutation Noise2noise denoising (SPEND)

We employed SPEND^18^ to achieve about eight times signal-to-noise ratio (SNR) enhancement of raw hyperspectral data. A four-layer U-Net deep learning model was trained using fourty hyperspectral datasets. Training and prediction were conducted on an Nvidia RTX 4090 GPU with 28GB of memory, requiring approximately 30 min for training and 20 s per stack for prediction. The SNR can be improved by ten times after denoising.

### Yeast maintenance and strains

For Htt103Q-GFP, yeast cells were pre-cultured in synthetic defined medium lacking uracil (SD-ura) for 16 h. ~300 μL of cells were then transferred to SD-ura supplemented with estradiol for 24 h at 30 °C (shaking) to induce protein expression^9^. In the initial experiment, an estradiol concentration of 100 nM was used. For the replication experiment, a higher concentration of 200 nM was applied to ensure induction efficiency. The results were consistent across both conditions, confirming that the aggregate formation is complete under both conditions. This estradiol induction facilitated the formation of Htt aggregates within the cells, providing a consistent model for studying stable protein aggregates. Cells were imaged the next day.

For α-synuclein-YFP, yeast cells were initially pre-cultured at 30 °C (shaking) for 16 h in yeast peptone dextrose (YPD) broth for plasmid selection. Yeast samples were placed into a centrifuge to pellet the cells (~2000 × *g* for 5 min). Supernatant was removed and the pellet was resuspended in sterile water for a washing step (repeated 3× times). After the final wash, cells were then transferred to Yeast Peptone (YP) medium containing 2% raffinose as the sole carbon source for 24 h, allowing adaptation to a non-fermentable carbon source. After 8 h, the cells were pelleted and washed with water 3× times, and were then added to YP + 2% galactose for 8 h (overnight) and grown at 30 °C (shaking). This incubation induced α-synuclein expression and condensate formation. Cells were then imaged the next day.

### Yeast mounting and imaging

1 mL of induced yeast samples were placed into a 1.5 mL Eppendorf tube and then centrifuged to pellet the cells (~1000 × *g* for 1 min). Supernatant was removed and the pellet was resuspended in sterile water for a washing step (repeated 3× times). After the final wash, a small amount of water was left to resuspend the pellet in (~150 μL). Two microliters of yeast was then pipetted onto a 24 × 60 mm^2^ #1 thickness cover slide. A calcium fluoride (CaF_2_) disk was then lowered onto the samples and fixed in place with tape. Yeast was imaged with CaF_2_ face down to allow the IR pulses to hit samples without glass absorption. Yeast was imaged with a pulsed IR frequency of 200 kHz and a pulse width of either 200 ns (Htt) or 400 ns (α-synuclein). Visible 488 nm excitation of the fluorescent proteins used 400 kHz with a 30% duty cycle.

### Yeast data analysis

Regions of interest were selected using Fiji (ImageJ) ROI manager select tool on each hyperspectral stack. Regions of interest with distinct amide I and II bands were selected. A custom Macro was written to loop through the same ROI coordinate in both the denoised FILM image and the DC fluorescence image stack (si_code_1.ijm). Spectra were then analyzed in RStudio (si_code_2.R). First, an offset is applied to bring the smallest value’s baseline to 0. Next, the FILM\ signal was divided by the DC fluorescence signal to account for bleaching of the fluorophore. All spectra were then divided by the IR laser power and then normalized by area under the curve (AUC), which is presented in the paper as the finalized calibrated spectrum. A rolling average (window, *k* = 5) was applied to smooth the spectrum.

### *C. elegans* maintenance and strains

AM141[unc-54p::Q40::YFP] (40 poly-glutamine fused to fluorescent yellow protein) was used for studying aggregates. Aggregates develop as the worm ages from diffuse protein. glh-1(sam24[glh-1::GFP::3xFLAG)] was used for studying condensates in *C. elegans* embryos. AM141 worms were synchronized and grown at 15 °C. Worms were collected from the same plate at larval stage 3 (L3), day 1 adults, or day 2 adults. In one experiment, we followed a single synchronized population over time, collecting animals at L3, then at adult day 1, and against at adult day 2. In the other two experiments, we paired L3 with a single adult stage—either L3 with day 1 adult, or L3 with day 2 adult—using animals taken from the same synchronized population. Day 1 adult GLH::GFP worm embryos were synchronized and imaged across two experiments to obtain two biological replicates.

Worms were cultured on nematode growth medium (for 1 L: phosphate buffer 20 mM, CaCl2 0.8 mM, MgSO4 0.8 mM, agar 17.5 g, NaCl 3.0 g, peptone 2.5 g, cholesterol 0.005 g). Plates were seeded with OP-50 *Escherichia coli*. To synchronize worm populations, 1 mL of 5 N NaOH and 0.5 mL of bleach were added to 3.5 mL sterile water containing embryos and gravid adults. The synchronized population was grown at 15 °C.

### *C. elegans* mounting and imaging

Worms were mounted via picking into 1% NaN_3_ in M9 buffer on a 24 × 60 mm^2^ #1 thickness cover slide. A calcium fluoride (CaF_2_) disk was then lowered onto the worms and fixed in place with tape. Worms were imaged with CaF_2_ face down to allow the IR pulses to hit samples without absorption. Worms were imaged with a pulsed IR frequency of 200 kHz and a pulse width of either 400 or 500 ns. Visible 488 nm excitation of the fluorescent proteins used 400 kHz with a 30% duty cycle.

### *C. elegans* data analysis

Regions of interest were selected using Fiji (ImageJ) ROI manager select tool on each hyperspectral stack. Regions of interest with distinct amide I and II bands were selected. A custom Macro was written to loop through the same ROI coordinate in both the denoised FILM image and the DC fluorescence image stack (si_code_1.ijm). Spectra were then analyzed in RStudio (si_code_2.R). First, an offset is applied to bring the smallest value’s baseline to 0. Next, the FILM signal was divided by the DC fluorescence signal to account for bleaching of the fluorophore. All spectra were then divided by the IR laser power, and then normalized by area under the curve (AUC), which is presented in the paper as the finalized calibrated spectrum. A rolling average (window, *k* = 5) was applied to smooth the spectrum.

### *C. elegans* fluorescent recovery after photobleaching (FRAP)

AM141 Q40::YFP aggregate worms were grown to the appropriate age at either 15 or 21 °C on NGM plates, as in the FILM experiments. Young adult GLH-1::GFP condensate worms were grown at 15 °C and dissected for embryonic P-granule FRAP. For imaging, three layers of nail polish were used to create an enclosed rectangle on a standard microscope slide. Aggregate worms were mounted in 1% NaN_3_ in M9 buffer. P-granule worms were dissected in 1× ex vivo buffer (25 mM HEPES pH 7, 150 mM NaCl, 2 mM CaCl_2_, 2 mM MgCl_2_)^5^. A #1.5 thickness cover slide was then affixed over the sample and sealed with more nail polish.

For scanning, samples were loaded onto a Nikon Ti microscope with a Nikon C2plus Si laser scanning confocal operated in filter mode with a 15 mW 488 nm laser diode for excitation and conventional GFP emission filters. The confocal aperture was set to 1, and 512 × 512 size for all scans. Using either a Plan Apo λ 20× lens with 0.8 NA (PolyQ) or a water-immersion Plan Apo λs 40x lens with 1.3 NA (P-granule condensates), the samples were selected visually by epifluorescence and then scanned. After the background images were acquired, the scan area was zoomed to just the region of interest for bleaching. Laser power was raised for the bleaching phase. Both the zoom and laser power were lowered back to initial value for the recovery monitoring. The conditions of bleaching varied by sample as described below. Recovery was always recorded at 5 s intervals using 1.08% laser power

For L3 PolyQ aggregates, bleaching 5% laser power, 10 s duration.

For day 1 PolyQ aggregates, bleaching 10% laser power 20 s duration.

For day 2 PolyQ aggregates, bleaching 10% laser power 25 s duration.

For condensates (40×), bleaching 5% laser power 5 s duration.

### Reporting summary

Further information on research design is available in the Nature Portfolio Reporting Summary linked to this article.

## Supplementary information


Transparent Peer Review file
Supplementary Fig. 1 and 2
Reporting summary


## Data Availability

Data, including raw and SPEND-denoised hyperspectrals, and fluorescent images, are available in zipped format on FigShare (https://doi.org/10.6084/m9.figshare.31385239). Numerical source data for all main figures and supplemental codes used for data analysis (si_code_1.ijm and si_code_2.R) can also be found on FigShare. Any additional data are available upon reasonable request to the authors.

## Code availability

A custom ROI loop ImageJ Macro has been provided as si_code_1.ijm, which allows users to loop through their DC fluorescence images and FILM hyperspectral images to obtain Mean Gray intensities for use in the R code. A custom R Studio data analysis code has also been provided (si_code_2.R.). The R code can be used to take files generated with the ImageJ Macro and IR power spectra and convert it into calibrated FILM spectra. In addition, it allows for a comparison between multiple conditions, generating the plots and box-and-whisker plots shown in Figs. 2 and 3. Written code and clean-up/commenting were assisted with ChatGPT.

## References

[1] Ross, C. A. & Poirier, M. A. Protein aggregation and neurodegenerative disease. *Nat. Med.* **10**, S10–S17 (2004). 15272267 10.1038/nm1066

[2] Banani, S. F., Lee, H. O., Hyman, A. A. & Rosen, M. K. Biomolecular condensates: organizers of cellular biochemistry. *Nat. Rev. Mol. Cell Biol.* **18**, 285–298 (2017). 28225081 10.1038/nrm.2017.7 PMC7434221

[3] Kenworthy, A. K. What’s past is prologue: FRAP keeps delivering 50 years later. *Biophys. J.* **122**, 3577–3586 (2023). 37218127 10.1016/j.bpj.2023.05.016 PMC10541474

[4] Morley, J. F., Brignull, H. R., Weyers, J. J. & Morimoto, R. I. The threshold for polyglutamine-expansion protein aggregation and cellular toxicity is dynamic and influenced by aging in Caenorhabditis elegans. *Proc. Natl. Acad. Sci. USA* **99**, 10417–10422 (2002). 12122205 10.1073/pnas.152161099 PMC124929

[5] Putnam, A., Cassani, M., Smith, J. & Seydoux, G. A gel phase promotes condensation of liquid P granules in Caenorhabditis elegans embryos. *Nat. Struct. Mol. Biol.* **26**, 220–226 (2019). 30833787 10.1038/s41594-019-0193-2 PMC6668929

[6] Shivu, B. et al. Distinct β-sheet structure in protein aggregates determined by ATR–FTIR spectroscopy. *Biochemistry* **52**, 5176–5183 (2013). 23837615 10.1021/bi400625v

[7] Hess, N., & Joseph, J. A. Structured protein domains enter the spotlight: modulators of biomolecular condensate form and function. *Trends Biochem. Sci.* **50**, 206–223 (2025). 10.1016/j.tibs.2024.12.008 PMC12165725 39827079

[8] Klementieva, O. et al. Super-resolution infrared imaging of polymorphic amyloid aggregates directly in neurons. *Adv. Sci.* **7**, 1903004 (2020). 10.1002/advs.201903004 PMC7080554 32195099

[9] Guo, Z. et al. Structural mapping of protein aggregates in live cells modeling Huntington’s disease. *Angew. Chem. Int. Ed.* **136**, e202408163 (2024). 10.1002/anie.202408163 PMC11781839 38880765

[10] Ao, J. et al. FILM: mapping organellar metabolism by mid-infrared photothermal-modulated fluorescence. *Nat. Methods.*10.1038/s41592-026-03090-1 (2026).42098345

[11] Guo, Z., Chiesa, G., & Cheng, J. X. Feeling the vibes: vibrational spectroscopic imaging of biomolecular assemblies in their natural environment. *Appl.**Phys. Rev.* **12**, 041312 (2025). 10.1063/5.0244025 PMC13196607 42183478

[12] Sun, R., Zhuang, Y., Lin, Y. & Hu, F. In situ secondary structure imaging of protein phase separation and aggregation by hyperspectral stimulated Raman scattering microscopy. *Nat. Commun.* **16**, 8552 (2025). 41022842 10.1038/s41467-025-63894-1 PMC12479984

[13] Wang, H. et al. Bond-selective fluorescence imaging with single-molecule sensitivity. *Nat. Photonics* **17**, 846–855 (2023). 38162388 10.1038/s41566-023-01243-8 PMC10756635

[14] Zhang, Y. et al. Fluorescence-detected mid-infrared photothermal microscopy. *J. Am. Chem. Soc.* **143**, 11490–11499 (2021). 34264654 10.1021/jacs.1c03642 PMC8750559

[15] Li, M. et al. Fluorescence-detected mid-infrared photothermal microscopy. *J. Am. Chem. Soc.* **143**, 10809–10815 (2021). 34270255 10.1021/jacs.1c03269

[16] Samolis, P., Zhu, X., & Sander, M. Y. Boxcar gating for time-resolved mid-infrared photothermal imaging of axon-bundle water boundaries. In *Proc.**CLEO Conference 2023* (Optica, Technical Digest Series, 2023).

[17] Yin, J. et al. Video-rate mid-infrared photothermal imaging by single-pulse photothermal detection per pixel. *Sci. Adv.* **9**, eadg8814 (2023). 37315131 10.1126/sciadv.adg8814 PMC10266719

[18] Ding, G. et al. Self-supervised elimination of non-independent noise in hyperspectral imaging. *Newton* **1**, 100195 (2025). 10.1016/j.newton.2025.100195 PMC12345349 40814694

[19] Garcia, S. M., Casanueva, M. O., Silva, M. C., Amaral, M. D. & Morimoto, R. I. Neuronal signaling modulates protein homeostasis in Caenorhabditis elegans post-synaptic muscle cells. *Genes Dev.* **21**, 3006–3016 (2007). 18006691 10.1101/gad.1575307 PMC2049200

[20] Chen, W. et al. The dynamics of P granule liquid droplets are regulated by the Caenorhabditis elegans germline RNA helicase GLH-1 via its ATP hydrolysis cycle. *Genetics* **215**, 421–434 (2020). 32245789 10.1534/genetics.120.303052 PMC7268986

