# Supplementary material for: Resolving protein condensates and aggregates in vivo by boxcar-enhanced Fluorescence-detected mid-Infrared photothermaL Microscopy (FILM)
Source: Commun Chem. 2026 May 11;9:287. doi: 10.1038/s42004-026-01970-3 (PMC13522537; doi:10.1038/s42004-026-01970-3)
Supplement: Supplementary file 1 [file 42004_2026_1970_MOESM1_ESM.pdf]

nature portfolio

Peer Review File

Resolving Protein Condensates and Aggregates in vivo by Boxcar-Enhanced Fluorescence-detected Mid-Infrared PhotothermaL Microscopy (FILM)

Corresponding Author: Professor Ji-Xin Cheng

This file contains all reviewer reports in order by version, followed by all author rebuttals in order by version.

Version 0:

Reviewer comments:

Reviewer #1

(Remarks to the Author)
In this manuscript Weinberg, Guo and co-authors apply box-car detected fluorescence-detected PTIR imaging to protein structure characterization is a couple of biological systems - yeast cells with Huntington's disease gene, and C.elegans. In my opinion, the approach that authors take is sound, statistical analysis looks solid, and the results are pretty interesting to be published. The main weakness of the manuscript is, in my opinion, its pretty incremental nature - all of the instrumentation and data analysis strategies used here were already presented in separate manuscripts by the same group, plus the results look pretty derivative from the group's previous paper that already applied F-PTIR to the same HD yeast cell system (https://doi.org/10.1002/anie.202408163). Since both the method and the application have been demonstrated by the group before, I believe that the following should be addressed before this manuscript can be published:

1. The authors are encouraged to additionally highlight the novelty aspect of the work and provide a clearer explanation of the advances made in this manuscript compared to the previous paper (https://doi.org/10.1002/anie.202408163).

2. The literature review could be more comprehensive. In particular, Dr. Simpson's F-PTIR work was not cited alongside the other F-PTIR demonstration paper (https://doi.org/10.1021/jacs.1c03269), the commercial implementation was not cited (https://doi.org/10.1021/acs.jmedchem.2c01359). Also, I think conceptually similar BonFIRE work on live cells by Dr. Wei should have been mentioned as well.

3. I would suggest to add a bit more details on the instrumentation and BoxCAR implementation rather than referencing another work for details. In particular, how wide was the BoxCAR window and how it was temporally positioned relative to the IR pulse? I believe this information is critical to make an assessment on thermal diffusion length and actual F-PTIR resolution.

4. I believe that the manuscript could benefit from a more detailed explanation on statistical analysis. In comparison between aggregates and condensates and different elegans ages how many different samples were prepared? How many replicates were made? The authors are encouraged to demonstrate the persistence of the observed change over multiple sample batches to suggest causation rather than correlation.

5. Providing a short summary of the de-noising algorithm rather than referencing previous work could be beneficial for readability of the manuscript.

6. What was the source of noticeable background fluorescence in Fig. 1b?

7. How much laser power was measured at the sample plane? The authors used a pretty short wavelength excitation source, so an assessment of potential phototoxicity to live samples could strengthen the case.

Reviewer #2

(Remarks to the Author)

This work represents an important advancement in the field of biospectroscopy, offering a valuable contribution by enabling the direct detection and discrimination of protein aggregates and condensates through vibrational imaging of secondary structures. The ability to perform such analyses in situ, while preserving the physiological context, provides critical insight into the structural dynamics of proteins involved in both health and disease.

In future work, the field would benefit from efforts to further bridge the spectroscopic signatures with actual structural models of protein assemblies. Attempts to interpret the detected secondary structure features in terms of real 3D conformations—possibly through correlation with cryo-electron microscopy or other high-resolution structural methods—would greatly enhance the impact of this approach and further solidify its role in structural biology.

Reviewer #3

(Remarks to the Author)
Resolving Protein Condensates and Aggregates in vivo by Optical Boxcar-enhanced Fluorescence-detected Mid-infrared Photothermal Microscopy

Summary:
This manuscript describes optical-boxcar fluorescence-detected mid-infrared photothermal (OBF-MIP) microscopy. The key innovations of the manuscript appear to be centered on: i) the demonstration of OBF-MIP as a new modality, and ii) the application of OBF-MIP to overcome limitations of FRAP for discrimination between aggregates vs. condensates in a model C. elegans system. Specifically, the authors hypothesize that subtle differences in the amide vibrational absorption spectrum can serve as spectroscopic signatures for differentiating between aggregates and condensates in vivo. Notably, this work definitively provides the first direct spectroscopic evidence of protein conformational changes in vivo between protein condensates vs. aggregates, with implications for modeling and understanding pathological protein/p[protein interactions within living microenvironments. Overall, this manuscript is recommended for publication following minor revisions summarized below.
Suggested revisions:

1. Comparison / benchmarking with FRAP. The authors describe well both the strengths and limitations of FRAP for differentiating between aggregates and condensates, with negligible recovery occurring in aggregates and relatively fast recovery within condensates. Given the historic success of FRAP in this role and the use of fluorescently labeled proteins, the manuscript would be significantly strengthened by additional studies designed specifically to benchmark the capabilities of OBF-MIP against FRAP within the same biological system.

2. Looked only at systems with well-established behavior. The novelty of the new knowledge gained from these studies should be better stated and emphasized. The aggregation propensity of AM141 with YFP/GFP labeling in C. elegans as a model system is well established. Furthermore, the relative increase in beta-sheet character within the IR absorption of proteins upon aggregation is also well-known. It appears that the main innovation in the current work is to definitively connect these two dots by enabling IR spectroscopy in vivo to support this hypothesis. What broader conclusions can we now draw with this in place, or conversely what hypotheses can be rejected based on this information?

3. The authors state that OBF-MIP is a novel method. While technically true, as measurements performed in this specific manner have not been reported previously, this work bears strong similarities to optical box-car photothermal imaging studies reported previously by the Sander group (also at Boston University), such as Anal. Chem., 2023, 95, 16514. The authors are encouraged to clarify the novelty of this method within the context of prior related work.

4. The authors employ the SPEND approach for sample denoising, then base their statistical analyses on the post-treated data. In this case, the spectral analysis has the potential to be interdependent on the spatial denoising, which likely implicitly assumes a noise distribution within the denoising algorithm. The authors are encouraged to perform the spectral analysis (which is aggregated in the statistics in any case) directly on the raw measurements rather than on the denoised data to minimize the possible introduction of spatial/spectral biases, or alternatively include additional more detailed simulations within the SI validating the absence of spectral bias from denoising of spatially structured samples.

5. The right-most images in figure 1b carry redundant information with those to their left and are not used within the manuscript narrative.

Version 1:

Reviewer comments:

Reviewer #3

(Remarks to the Author)
Dear Editor,

Please find below comments on the revised manuscript by Wang, Cheng, and coworkers. The authors have made changes to the manuscript to address several of the original comments. However, some additional suggested minor revisions are recommended prior to publication, details of which are provided below.

1. The authors have made significant changes to the manuscript, including a direct comparison between the box-car MIP and conventional FRAP, highlighting the comparable information content available from the two methods. No additional revisions are recommended.

2. The authors have made no changes to the manuscript in response to the request for additional clarification on the novelty of this study relative to their previous publication on optical box-car fluorescence (OBF) MIP. In their response, the authors highlight the significance of their in vivo analysis capabilities, enabling the tracking of age-dependent onset of aggregation effects. However, this point does not appear in the Abstract as a key finding, nor in the Introduction as a key motivation. Both are more focused on the technique itself, which the authors have published on previously. The authors are encouraged to update the Abstract and Introduction to more properly align them with the key innovations claimed for the present study.

3. Again, the authors are encouraged to update the manuscript to, at the very least, include a citation to the prior work by the Sander group on boxcar MIP and a short paragraph highlighting the distinctions between them, given the potential for confusion among readers familiar with photothermal microscopy methods. It does not appear that any such reference appeared in their prior work on OBF-MIP (ref. #10) either, such that the potential for confusion has not been resolved previously. Although the authors state to have added additional details to the Methods section to improve clarity, no obvious changes were marked up in the revised manuscript.

4. The authors have made changes to the Introduction to better clarify the mechanism of action for implementation for the SPEND denoiser. It is not clear to this reviewer that spectral bias from spatial/spectral correlations has been suppressed, but the SPEND algorithm is arguably not the core focus of the present study. Additional revisions are not required.

5. Suggested revisions to Figure 1 have been implemented.

Open Access This Peer Review File is licensed under a Creative Commons Attribution 4.0 International License, which permits use, sharing, adaptation, distribution and reproduction in any medium or format, as long as you give appropriate credit to the original author(s) and the source, provide a link to the Creative Commons license, and indicate if changes were made.
In cases where reviewers are anonymous, credit should be given to 'Anonymous Referee' and the source.
The images or other third party material in this Peer Review File are included in the article’s Creative Commons license, unless indicated otherwise in a credit line to the material. If material is not included in the article’s Creative Commons license and your intended use is not permitted by statutory regulation or exceeds the permitted use, you will need to obtain permission directly from the copyright holder.
To view a copy of this license, visit https://creativecommons.org/licenses/by/4.0/

Reviewers' comments:

Reviewer #1 (Remarks to the Author):

In this manuscript Weinberg, Guo and co-authors apply box-car detected fluorescence-detected PTIR imaging to protein structure characterization is a couple of biological systems - yeast cells with Huntington's disease gene, and C.elegans. In my opinion, the approach that authors take is sound, statistical analysis looks solid, and the results are pretty interesting to be published. The main weakness of the manuscript is, in my opinion, its pretty incremental nature - all of the instrumentation and data analysis strategies used here were already presented in separate manuscripts by the same group, plus the results look pretty derivative from the group's previous paper that already applied F-PTIR to the same HD yeast cell system (https://doi.org/10.1002/anie.202408163). Since both the method and the application have been demonstrated by the group before, I believe that the following should be addressed before this manuscript can be published:

Response: We thank the reviewer for taking the time to read our manuscript! We hope that we have emphasized the novelty of the technique in contrast to pre-existing methods, as well as answered the remaining questions sufficiently below.

1. The authors are encouraged to additionally highlight the novelty aspect of the work and provide a clearer explanation of the advances made in this manuscript compared to the previous paper (https://doi.org/10.1002/anie.202408163).

Response: We expand on previous fluorescently-guided mid-infrared microscopy by demonstrating the acquisition of hyperspectral stacks that can be used without deconvolution for secondary structure interpretations. Our work further demonstrates OBF-MIP's versatility in both quantitively distinguishing aggregates (even early, fluid aggregates) from condensates, as well as enabling a more nuanced view of secondary structure heterogeneity across aging aggregates and condensates. We suggest that OBF-MIP can become a key method in the toolbox for studying condensates, aggregates, and other protein assemblies, and provide techniques and workflows to enable that study.

Although the instrumentation and data analysis strategies have been also reported in our preprint, the application of this technology to assess the full continuum of protein states is entirely new in this manuscript. In particular, our study uncovers a previously unrecognized progression from liquid-like protein condensates to solid protein aggregates during aging. Distinct from our earlier work that focused exclusively on mature aggregates, the current manuscript presents the first chemical imaging of protein condensates using OBF-MIP.

With the ability to capture the whole spectrum of protein states and quantify their changes, we show that pathological Huntington's disease proteins initially form liquid-like condensates and then progressively transition into aggregates during aging.

This newly revealed progression demonstrates the unique power of our method in tracking protein states in both time and space and within their native context. As commented by Reviewer 3, our work "provides the first direct spectroscopic evidence of protein conformational changes *in vivo* between protein condensates vs. aggregates, with implications for modeling and understanding pathological protein/protein interactions within living microenvironments."

Therefore, we believe this work goes well beyond incremental advance and establish an important new application of OBF-MIP for protein physical biology.

2. The literature review could be more comprehensive. In particular, Dr. Simpson's F-PTIR work was not cited alongside the other F-PTIR demonstration paper (https://doi.org/10.1021/jacs.1c03269), the commercial implementation was not cited (https://doi.org/10.1021/acs.jmedchem.2c01359). Also, I think conceptually similar BonFIRE work on live cells by Dr. Wei should have been mentioned as well.

Response: We thank the reviewer for the suggestions. Dr. Simpson's F-PTIR paper has been cited into the introduction, along with BONFire. The key difference with Li et al. 2021 is that our work enables *in vivo* live imaging of aqueous biological samples using transgenic animals. Our work also expands on F-PTIR by reducing bleaching using the optical boxcar method, in addition to using AI SPEND denoising to further refine spectra. BonFIRE, while similar in that it uses IR and fluorescence, fundamentally differs from our method in that it is reliant on dyes and cannot be applied to pre-existing transgenic animals, nor has it provided evidence in its ability to differentiate secondary structure.

3. I would suggest to add a bit more details on the instrumentation and BoxCAR implementation rather than referencing another work for details. In particular, how wide was the BoxCAR window and how it was temporally positioned relative to the IR pulse? I believe this information is critical to make an assessment on thermal diffusion length and actual F-PTIR resolution.

Response: We thank the reviewer for the suggestion. Additional information on optical BoxCAR has been added to the introduction and Fig. 1, including the frequency used for both the fluorescent probe and IR excitation. In our OBF-MIP implementation, we modulate the visible beam (either the fluorescence-excitation beam or the probe beam in the

scattering-based scheme) into pulses at twice the IR repetition rate. Please also see the remarks to reviewer #3 below, in response 4, which compare and contrast to work done by the Sander's group.

4. I believe that the manuscript could benefit from a more detailed explanation on statistical analysis. In comparison between aggregates and condensates and different elegans ages how many different samples were prepared? How many replicates were made? The authors are encouraged to demonstrate the persistence of the observed change over multiple sample batches to suggest causation rather than correlation.

Response: We appreciate the reviewer's suggestion and have added additional replicates for both yeast and worm samples. For the yeast data, two biologically independent experiments were conducted on htt103Q and α-synuclein. For the worm data, two biologically independent replicates are included for PolyQ aggregates. In one experiment, we followed a single synchronized population over time, collecting animals at L3, then at adult day 1, and against at adult day 2. In the other two experiments, we paired L3 with a single adult stage—either L3 with day 1 adult, or L3 with day 2 adult—using animals taken from the same synchronized population. For P-granule condensates, embryos were obtained from day 1 adults. The numbers of spectra used in the statistical analysis are now included in Figure legends.

5. Providing a short summary of the de-noising algorithm rather than referencing previous work could be beneficial for readability of the manuscript.

Response: Based on the reviewer's suggestion, a summary was provided in the introduction. Briefly, SPEND uses even and odd spectral frames to generate two stacks with the same levels of noise. These two stacks can then be used for self-supervised noise-to-noise training. Additional information on SPEND denoising impacts on spectra (as compared to raw) has been added to Fig. 1.

6. What was the source of noticeable background fluorescence in Fig. 1b?

Response: We thank the reviewer for bringing this to our attention. In the first column of fluorescent images, the background signal is likely due to fluorescently-labeled protein diffused throughout the cytosol, as not all proteins form aggregates/condensates. It is also possible that part of the background arises from autofluorescence, which is commonly observed in biological samples. In the last two column of denoised OBF-MIP images, diffused cytosolic proteins and autofluorescent signals may likely contribute to faint OBF-

MIP signals. We would like to clarity that our analyses focus exclusively on the protein states of condensates/aggregates, and do not include diffusive protein populations or autofluorescent signals.

7. How much laser power was measured at the sample plane? The authors used a pretty short wavelength excitation source, so an assessment of potential phototoxicity to live samples could strengthen the case.

Response: The visible laser power at the sample plane is 15 μW for P-granules and 1 μW for PolyQ. The mid-IR laser power is Wavenumber dependent, with a maximum of 2 mW. The visible excitation wavelength of 488 nm is chosen according to the fluorophore absorption spectrum, consistent with standard fluorescence microscopy practices.

Reviewer #2 (Remarks to the Author):

This work represents an important advancement in the field of biospectroscopy, offering a valuable contribution by enabling the direct detection and discrimination of protein aggregates and condensates through vibrational imaging of secondary structures. The ability to perform such analyses in situ, while preserving the physiological context, provides critical insight into the structural dynamics of proteins involved in both health and disease.

Response: We thank the reviewer for the positive comments of our method.

In future work, the field would benefit from efforts to further bridge the spectroscopic signatures with actual structural models of protein assemblies. Attempts to interpret the detected secondary structure features in terms of real 3D conformations—possibly through correlation with cryo-electron microscopy or other high-resolution structural methods—would greatly enhance the impact of this approach and further solidify its role in structural biology.

Response: We appreciate the reviewer's insight, and have included these perspectives in the discussion.

Reviewer #3 (Remarks to the Author):

Resolving Protein Condensates and Aggregates *in vivo* by Optical Boxcar-enhanced

Fluorescence-detected Mid-infrared Photothermal Microscopy

Summary:
This manuscript describes optical-boxcar fluorescence-detected mid-infrared photothermal (OBF-MIP) microscopy. The key innovations of the manuscript appear to be centered on: i) the demonstration of OBF-MIP as a new modality, and ii) the application of OBF-MIP to overcome limitations of FRAP for discrimination between aggregates vs. condensates in a model C. elegans system. Specifically, the authors hypothesize that subtle differences in the amide vibrational absorption spectrum can serve as spectroscopic signatures for differentiating between aggregates and condensates *in vivo*. Notably, this work definitively provides the first direct spectroscopic evidence of protein conformational changes *in vivo* between protein condensates vs. aggregates, with implications for modeling and understanding pathological protein/protein interactions within living microenvironments. Overall, this manuscript is recommended for publication following minor revisions summarized below.

Response: We thank the reviewer for taking the time to read this manuscript, and recommending its publication with minor revisions.

Suggested revisions:

1. Comparison / benchmarking with FRAP. The authors describe well both the strengths and limitations of FRAP for differentiating between aggregates and condensates, with negligible recovery occurring in aggregates and relatively fast recovery within condensates. Given the historic success of FRAP in this role and the use of fluorescently labeled proteins, the manuscript would be significantly strengthened by additional studies designed specifically to benchmark the capabilities of OBF-MIP against FRAP within the same biological system.

Response: We appreciate the reviewer's suggestion. Fig. 4 now contains a benchmark against FRAP in worm condensates and across three PolyQ aggregates. We show good agreement with the FRAP results, indicating that the PolyQ assemblies shown to contain high β-sheet with OBF-MIP display the slowest FRAP recovery, providing more evidence for our conclusions.

2. Looked only at systems with well-established behavior. The novelty of the new knowledge gained from these studies should be better stated and emphasized. The aggregation propensity of AM141 with YFP/GFP labeling in C. elegans as a model system is

well established. Furthermore, the relative increase in beta-sheet character within the IR absorption of proteins upon aggregation is also well-known. It appears that the main innovation in the current work is to definitively connect these two dots by enabling IR spectroscopy *in vivo* to support this hypothesis. What broader conclusions can we now draw with this in place, or conversely what hypotheses can be rejected based on this information?

Response: We thank the reviewer for this question. We agree with the reviewer that the aggregation propensity of YFP-labeled polyQ is established in *C. elegans* and increased beta-sheet character is a known hallmark of protein aggregation. The new advance of our work is to show when and how this aggregation propensity emerges *in vivo* during aging. Specifically, we show that at the larval stage of *C. elegans,* YFP-labeled polyQ assemblies predominantly exhibit condensate-like properties, and only with increasing age do they progressively acquire intermediate and ultimately aggregate-like structural features. Our data therefore provide direct, *in vivo* chemical evidence for a condensate to intermediate to aggregate progression with increasing age, a pathway that could not previously be visualized chemically within intact animals.

This finding rules out the possibility that early, large Huntington assemblies *in vivo* are already beta-sheet-rich microaggregates. In addition, by simultaneously imaging multiple protein assemblies within intact tissues, we show that their structural properties vary across cells, revealing that the maturation of aggregates is heterogeneous. This capability to quantitatively resolve protein states *in vivo* represents an important advancement.

3. The authors state that OBF-MIP is a novel method. While technically true, as measurements performed in this specific manner have not been reported previously, this work bears strong similarities to optical box-car photothermal imaging studies reported previously by the Sander group (also at Boston University), such as Anal. Chem., 2023, 95, 16514. The authors are encouraged to clarify the novelty of this method within the context of prior related work.

Response: We appreciate the valuable comment raised by the reviewer. In the work by the Sander group, the lock-in amplifier was replaced with a boxcar averager to extract the photothermal signal in the time domain rather than the frequency domain. This approach enhances the photothermal signal because photothermal excitation is a low-duty-cycle process that contains rich high-harmonic components. These components can be captured by a boxcar averager but may be lost when using a lock-in amplifier, which primarily detects the fundamental frequency unless additional instrumentation changes are made.

In our OBF-MIP implementation, we modulate the visible beam (either the fluorescence-excitation beam or the probe beam in the scattering-based scheme) into pulses at twice the IR repetition rate. In this configuration, the visible light serves as a carrier wave that shifts the higher odd-order photothermal harmonics into the fundamental frequency band detectable by a lock-in amplifier (Ao et al. 2025). Therefore, rather than replacing the detector as proposed by the Sander group from the electrical-detection side, we engineer the optical excitation itself by converting continuous-wave light into pulses. This inherently shifts the higher-order components into the detectable region while simultaneously reducing photo-dosage to the sample—an important advantage for mitigating fluorescence photobleaching.

To improve clarity, we have added more detailed explanations in the Methods section.

4. The authors employ the SPEND approach for sample denoising, then base their statistical analyses on the post-treated data. In this case, the spectral analysis has the potential to be interdependent on the spatial denoising, which likely implicitly assumes a noise distribution within the denoising algorithm. The authors are encouraged to perform the spectral analysis (which is aggregated in the statistics in any case) directly on the raw measurements rather than on the denoised data to minimize the possible introduction of spatial/spectral biases, or alternatively include additional more detailed simulations within the SI validating the absence of spectral bias from denoising of spatially structured samples.

Response: Thank you for this insightful comment. We agree that it is important to ensure that SPEND denoising does not introduce spatial or spectral bias into downstream statistical analyses. To address this, we have expanded the description of SPEND in the Introduction and added a direct comparison between raw and denoised spectra in Fig. 1, demonstrating that SPEND preserves spectral fidelity.

In this project, SPEND does **not** assume an explicit description of spatial noise model (e.g., a defined correlation length). Instead, it only assumes that adjacent frames in the spectral axis share statistically similar noise properties. This assumption holds because the noise characteristics are primarily determined by acquisition parameters—such as pixel dwell time and lock-in amplifier settings—which remain constant throughout the experiment. These conditions maintain high robustness and prevent distortion of spatial or spectral features by SPEND.

The SNR of the raw data is too low to provide high quality statistical analysis. Regarding the suggestion to validate spectral bias through simulations: as the spatially correlated noise in our system is hard to depict in a rigorous analytical form, generating realistic synthetic datasets is challenging.

Moreover, the fidelity of a denoiser is generally governed by the input SNR and the complexity of the features to be recovered, rather than by the imaging modality itself. The fidelity of SPEND has already been extensively validated in Ding *et al.* (2025), where SPEND accurately recovered both fine cellular spatial structure and Raman spectral features at input SNR as low as 9 (Fig. 3 and SI Fig. S12–S13). In the present study, the input SNR (>12) and target spectral and spatial features are substantially simpler, further reducing the likelihood of denoising-induced artifacts.

Taken together, these results support that SPEND maintains spectral integrity for the data used in this work.

5. The right-most images in figure 1b carry redundant information with those to their left and are not used within the manuscript narrative.
Response: We appreciate the reviewer's comment, and have removed the redundant image for ratiometric OBF-MIP images of yeast.

Please find below comments on the revised manuscript by Wang, Cheng, and coworkers. The authors have made changes to the manuscript to address several of the original comments. However, some additional suggested minor revisions are recommended prior to publication, details of which are provided below.

Response: We thank the reviewer for their additional comments and for taking time to read our updated manuscript.

1. The authors have made significant changes to the manuscript, including a direct comparison between the box-car MIP and conventional FRAP, highlighting the comparable information content available from the two methods. No additional revisions are recommended.

Response: We thank the reviewer for their suggestion to incorporate FRAP into the manuscript.

2. The authors have made no changes to the manuscript in response to the request for additional clarification on the novelty of this study relative to their previous publication on optical box-car fluorescence (OBF) MIP. In their response, the authors highlight the significance of their in vivo analysis capabilities, enabling the tracking of age-dependent onset of aggregation effects. However, this point does not appear in the Abstract as a key finding, nor in the Introduction as a key motivation. Both are more focused on the technique itself, which the authors have published on previously. The authors are encouraged to update the Abstract and Introduction to more properly align them with the key innovations claimed for the present study.

Response: We have added additional information to the abstract and to the introduction, highlighting our hypothesis/findings of age-related secondary structure changes in PolyQ aggregates.

3. Again, the authors are encouraged to update the manuscript to, at the very least, include a citation to the prior work by the Sander group on boxcar MIP and a short paragraph highlighting the distinctions between them, given the potential for confusion among readers familiar with photothermal microscopy methods. It does not appear that any such reference appeared in their prior work on OBF-MIP (ref. #10) either, such that the potential for confusion has not been resolved previously. Although the authors state to have added additional details to the Methods section to improve clarity, no obvious changes were marked up in the revised manuscript.

Response: We apologize for the oversight of not citing the Sander group for their innovation of electrical boxcar. We have added a comparison between electrical and optical boxcar in the introduction.

4. The authors have made changes to the Introduction to better clarify the mechanism of action

for implementation for the SPEND denoiser. It is not clear to this reviewer that spectral bias from spatial/spectral correlations has been suppressed, but the SPEND algorithm is arguably not the core focus of the present study. Additional revisions are not required.

Response: We thank the reviewer for their thoughts on SPEND.

5. Suggested revisions to Figure 1 have been implemented.

Response: We thank the reviewer for their suggestions!
