# Supplementary material for: Resolving protein condensates and aggregates in vivo by boxcar-enhanced Fluorescence-detected mid-Infrared photothermaL Microscopy (FILM)
Source: Commun Chem. 2026 May 11;9:287. doi: 10.1038/s42004-026-01970-3 (PMC13522537; doi:10.1038/s42004-026-01970-3)
Supplement: Supplementary file 2 [file 42004_2026_1970_MOESM2_ESM.pdf]

a

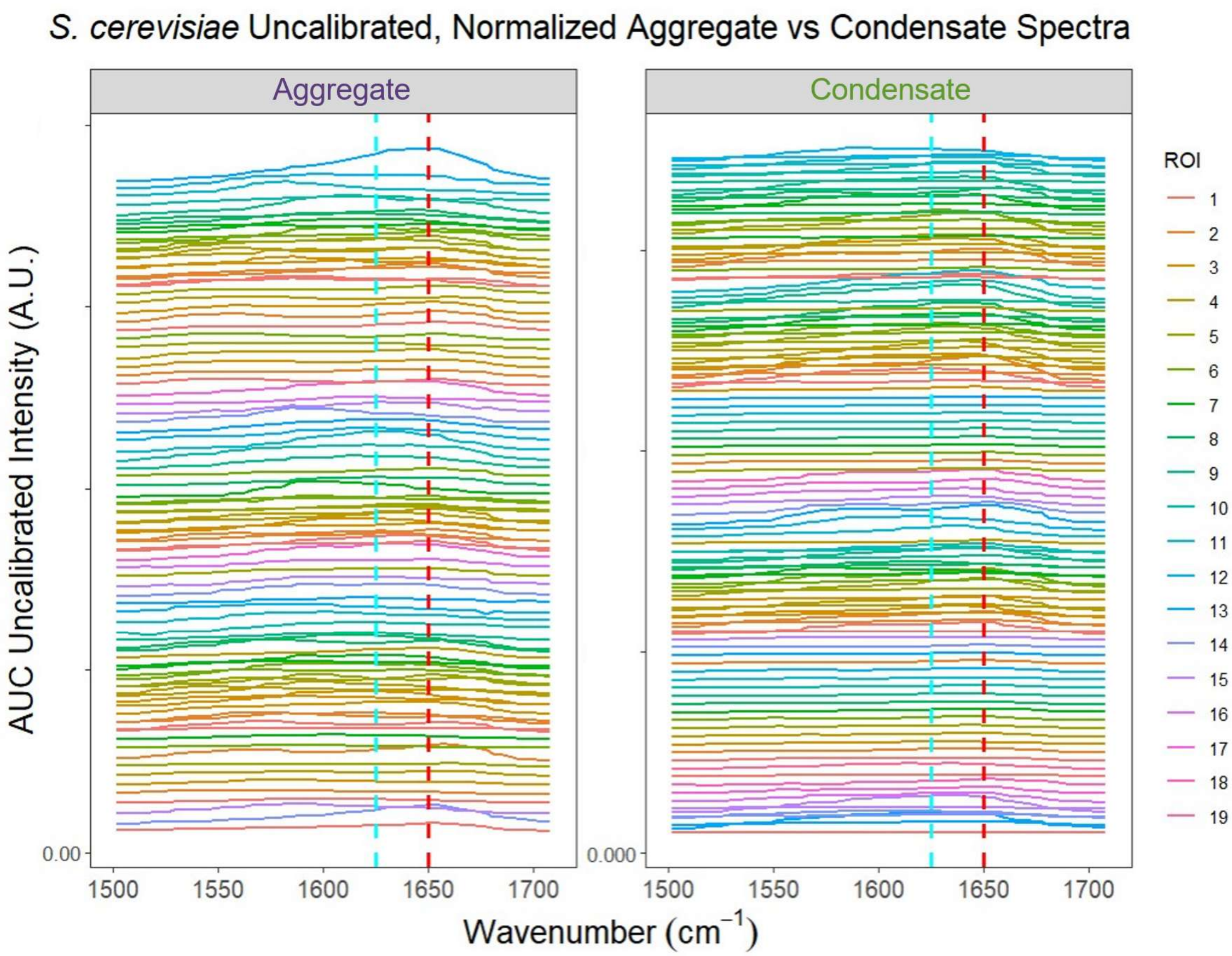

Supplemental Figure 1. a, SPEND-denoised but uncalibrated data of *S. cerevisiae* aggregates (htt108Q) and condensates (α-synuclein).

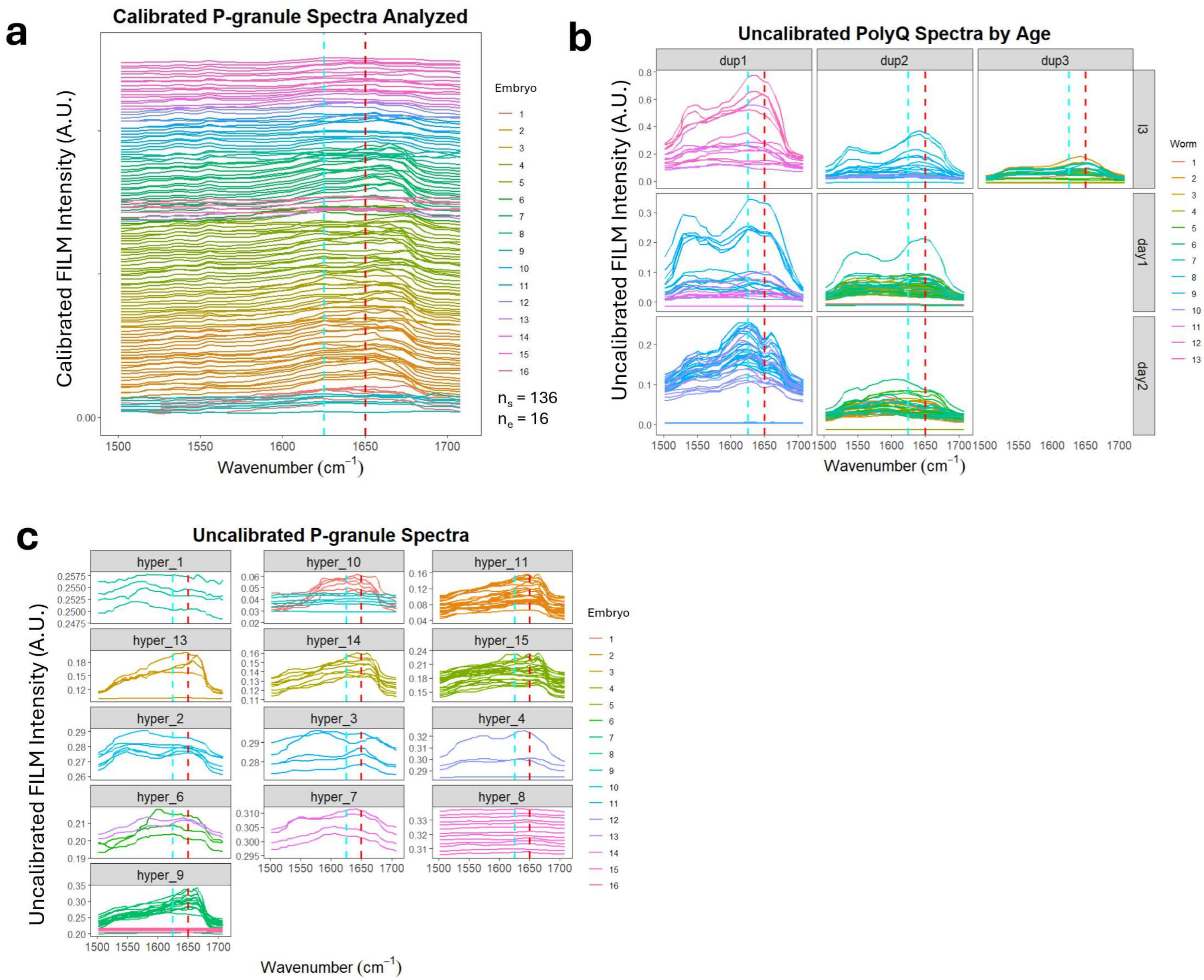

Supplemental Figure 2. a, Calibrated P granule spectra included in analysis. n_s = number of spectra. n_e = number of embryos. **b,** SPEND-denoised but uncalibrated data of *C. elegans* aggregates (PolyQ). **c,** SPEND-denoised but uncalibrated data of *C. elegans* condensates (P-granules).
