# Supplementary material for: Resolving protein condensates and aggregates in vivo by boxcar-enhanced Fluorescence-detected mid-Infrared photothermaL Microscopy (FILM)
Source: Commun Chem. 2026 May 11;9:287. doi: 10.1038/s42004-026-01970-3 (PMC13522537; doi:10.1038/s42004-026-01970-3)
Supplement: Supplementary file 3 [file 42004_2026_1970_MOESM3_ESM.pdf]

nature portfolio

Corresponding author(s): Ji-Xin Cheng, Meng Wang

Last updated by author(s): Feb 21, 2026

Reporting Summary

Nature Portfolio wishes to improve the reproducibility of the work that we publish. This form provides structure for consistency and transparency in reporting. For further information on Nature Portfolio policies, see our Editorial Policies and the Editorial Policy Checklist.

Statistics

For all statistical analyses, confirm that the following items are present in the figure legend, table legend, main text, or Methods section.

n/a	Confirmed	
☐	☒	The exact sample size (*n*) for each experimental group/condition, given as a discrete number and unit of measurement
☐	☒	A statement on whether measurements were taken from distinct samples or whether the same sample was measured repeatedly
☐	☒	The statistical test(s) used AND whether they are one- or two-sided<br>*Only common tests should be described solely by name; describe more complex techniques in the Methods section.*
☒	☐	A description of all covariates tested
☐	☒	A description of any assumptions or corrections, such as tests of normality and adjustment for multiple comparisons
☐	☒	A full description of the statistical parameters including central tendency (e.g. means) or other basic estimates (e.g. regression coefficient) AND variation (e.g. standard deviation) or associated estimates of uncertainty (e.g. confidence intervals)
☐	☒	For null hypothesis testing, the test statistic (e.g. *F*, *t*, *r*) with confidence intervals, effect sizes, degrees of freedom and *P* value noted<br>*Give P values as exact values whenever suitable.*
☒	☐	For Bayesian analysis, information on the choice of priors and Markov chain Monte Carlo settings
☒	☐	For hierarchical and complex designs, identification of the appropriate level for tests and full reporting of outcomes
☒	☐	Estimates of effect sizes (e.g. Cohen's *d*, Pearson's *r*), indicating how they were calculated

Our web collection on statistics for biologists contains articles on many of the points above.

Software and code

Policy information about availability of computer code

Data collection	LabView version 2024 was used for data collection.
Data analysis	Custom ImageJ/Fiji Macro and RStudio code was generated for this study, and are available in the Supplemental Information on FigShare.

For manuscripts utilizing custom algorithms or software that are central to the research but not yet described in published literature, software must be made available to editors and reviewers. We strongly encourage code deposition in a community repository (e.g. GitHub). See the Nature Portfolio guidelines for submitting code & software for further information.

Data

Policy information about availability of data

All manuscripts must include a data availability statement. This statement should provide the following information, where applicable:

- Accession codes, unique identifiers, or web links for publicly available datasets
- A description of any restrictions on data availability
- For clinical datasets or third party data, please ensure that the statement adheres to our policy

All data supporting the findings of this study are available within the paper, its supplementary Information, and on FigShare (doi.org/10.6084/m9.figshare.31385239)

Research involving human participants, their data, or biological material

Policy information about studies with human participants or human data. See also policy information about sex, gender (identity/presentation), and sexual orientation and race, ethnicity and racism.

Reporting on sex and gender	Research does not involve human participants, their data, or biological material.
Reporting on race, ethnicity, or other socially relevant groupings	Research does not involve human participants, their data, or biological material.
Population characteristics	Research does not involve human participants, their data, or biological material.
Recruitment	Research does not involve human participants, their data, or biological material.
Ethics oversight	Research does not involve human participants, their data, or biological material.

Note that full information on the approval of the study protocol must also be provided in the manuscript.

Field-specific reporting

Please select the one below that is the best fit for your research. If you are not sure, read the appropriate sections before making your selection.

☒ Life sciences ☐ Behavioural & social sciences ☐ Ecological, evolutionary & environmental sciences

For a reference copy of the document with all sections, see nature.com/documents/nr-reporting-summary-flat.pdf

Life sciences study design

All studies must disclose on these points even when the disclosure is negative.

Sample size	For yeast, over 70 spectra/individual yeast cells across two replicates were used for the study. For worms, at least 10 individual animals were used, and 16 individual embryos were used. Multiple spectra were obtained from each worm/embryo. n >= 10 was considered sufficient to demonstrate our technique for worms.
Data exclusions	Images were excluded from analysis if tissues displayed too much movement (due to heating). Spectra obtained from images were excluded only when the spectrum did not contain a clear, defined amide I and amide II peak (i.e., the spectrum did not contain key protein features).
Replication	Two replicates were performed for the yeast data, and 2-3 replicates were performed per age for the worm PolyQ data. Two replicates were performed for the worm embryos. All replication attempts were successful.
Randomization	Randomization not relevant to this study, as we were demonstrating differences between biological groups.
Blinding	Blinding was not necessary because subjective observations (i.e., morphology) was not a part of data analysis. No control/treatment groups were present.

Reporting for specific materials, systems and methods

We require information from authors about some types of materials, experimental systems and methods used in many studies. Here, indicate whether each material, system or method listed is relevant to your study. If you are not sure if a list item applies to your research, read the appropriate section before selecting a response.

Materials & experimental systems

n/a	Involved in the study	
☒	☐	Antibodies
☒	☐	Eukaryotic cell lines
☒	☐	Palaeontology and archaeology
☐	☒	Animals and other organisms
☒	☐	Clinical data
☒	☐	Dual use research of concern
☒	☐	Plants

Methods

n/a	Involved in the study	
☒	☐	ChIP-seq
☒	☐	Flow cytometry
☒	☐	MRI-based neuroimaging

Animals and other research organisms

Policy information about studies involving animals; ARRIVE guidelines recommended for reporting animal research, and Sex and Gender in Research

Laboratory animals	Saccharomyces cerevisiae: Htt103Q-GFP, α-synuclein-YFP. Caenorhabditis elegans: AM141 Q40::YFP (L3, day 1, day 2 adults), DUP64 GLH-1::GFP (day 1 adult embryos).
Wild animals	Study did not involve wild animals.
Reporting on sex	Hermaphrodite worms analyzed only; research question does not implicate sex-related differences.
Field-collected samples	Study does not involve samples collected from the field.
Ethics oversight	No ethical approval/guidance necessary for yeast and invertebrate experiments.

Note that full information on the approval of the study protocol must also be provided in the manuscript.

Plants

Seed stocks	*Report on the source of all seed stocks or other plant material used. If applicable, state the seed stock centre and catalogue number. If plant specimens were collected from the field, describe the collection location, date and sampling procedures.*
Novel plant genotypes	*Describe the methods by which all novel plant genotypes were produced. This includes those generated by transgenic approaches, gene editing, chemical/radiation-based mutagenesis and hybridization. For transgenic lines, describe the transformation method, the number of independent lines analyzed and the generation upon which experiments were performed. For gene-edited lines, describe the editor used, the endogenous sequence targeted for editing, the targeting guide RNA sequence (if applicable) and how the editor was applied.*
Authentication	*Describe any authentication procedures for each seed stock used or novel genotype generated. Describe any experiments used to assess the effect of a mutation and, where applicable, how potential secondary effects (e.g. second site T-DNA insertions, mosiacism, off-target gene editing) were examined.*
